# Why is the medical profession reluctant to talk about diet change?

**DOI:** 10.1016/j.fhj.2025.100231

**Published:** 2025-03-31

**Authors:** Shireen Kassam, Laura-Jane Smith

**Affiliations:** a King's College Hospital, London, England; b University of Winchester, Hampshire, England

**Keywords:** Vegan, Plant-based, Diet change, Nutrition, Food

## Abstract

The intertwined crises of poor health, climate change, biodiversity loss and social injustice demand urgent action. Human activities, particularly fossil fuel use and the current food system, are key drivers of these crises. A transition to a plant-based diet, especially within healthcare systems, offers a significant opportunity to address these challenges. Diets high in animal products and ultra-processed foods are leading causes of chronic ill health and environmental degradation, while plant-based diets reduce greenhouse gas emissions, conserve biodiversity and promote human health. Evidence shows that plant-based diets can prevent and manage conditions such as cardiovascular disease, type 2 diabetes and cancer, while addressing global food insecurity and resource inefficiency. Healthcare systems, such as the NHS, can lead this transition by offering plant-based meals, promoting education and advocating for policy changes. Embracing plant-based diets is now an ethical imperative, with benefits spanning individual health, environmental sustainability, equitable resource distribution and global health justice.

## Why is the medical profession reluctant to talk about diet change?

**Fig. 1:**
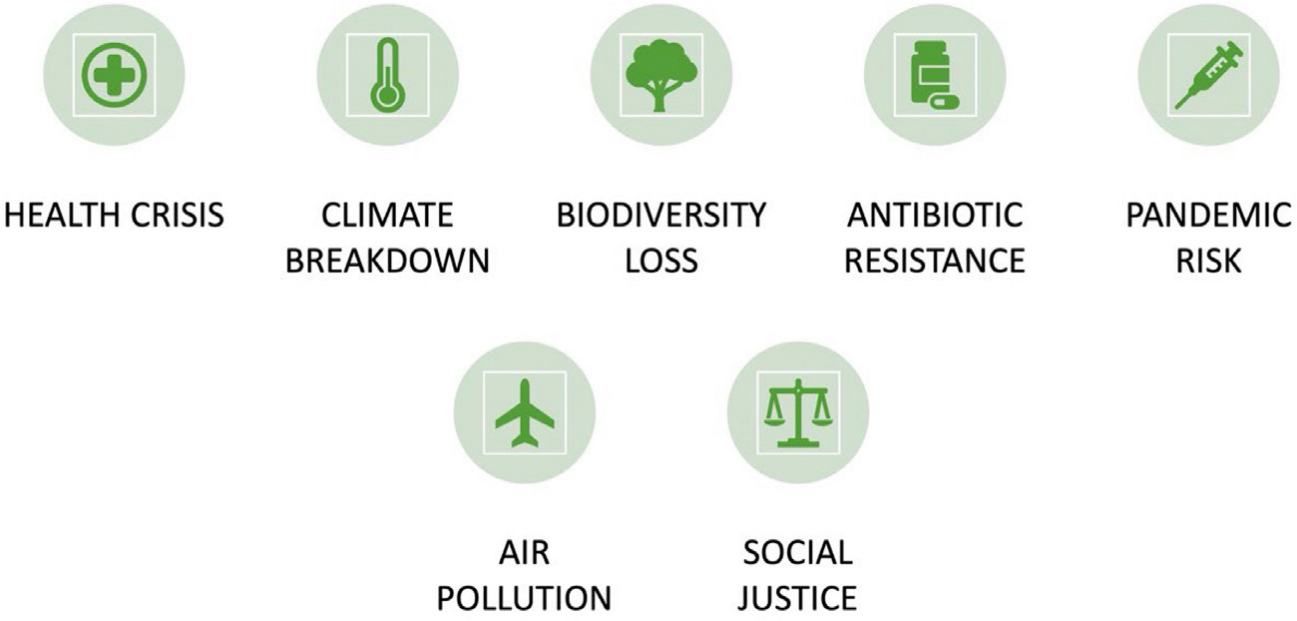
The multiple negative impacts of our current food system.

The world faces interconnected crises: poor health, climate breakdown, biodiversity loss and social inequity.^1^ No one is untouched, and the root causes lie in human actions. More people than ever are living in poor health, with low-income communities disproportionately affected. Among the primary drivers of this complex crisis are the continued use of fossil fuels and the global food system (Fig. 1). Tackling both is essential to achieving internationally recognised health, climate and environmental goals.^2^

Dietary risk factors are among the leading contributors to poor health.^3^ In addition, over 700 million people globally experience hunger, even though current food production could sustain >10 billion people.^1^^,^^4^ While health professionals are often at the forefront of advocacy against fossil fuel use, the same passion is rarely extended to dietary change. Yet, transitioning from animal-based agriculture to a plant-based food system offers transformative benefits. This shift aligns with a compassionate, inclusive approach to health – encompassing human, planetary and animal freedom – in line with the ‘One Health’ initiative.^5^

Despite the clear link between diet and health, a lack of nutrition education in both undergraduate and postgraduate medical training perpetuates harmful norms.^6^ Many feel constrained by concerns about patient choice, their own personal habits, or entrenched cultural and social norms. Our own research shows that dietitians face similar barriers, including a curriculum that has not kept pace with the science, prevailing myths, ingrained and often inaccurate beliefs, along with little support within the workplace to advocate for plant-based diets.^7^ Meanwhile, the food industry wields considerable influence, promoting meat and dairy as essential while falsely equating plant-based diets with ultra-processed foods.^8^ Green washing and misinformation tactics from animal farming groups mirror those used by the fossil fuel and tobacco industries, distorting public perception and delaying action.

## The UK’s food system: a mismatch with health goals

The UK’s agricultural landscape starkly illustrates the disconnect between policy and health. Currently, 85% of farmland is dedicated to raising animals for food. This leads to excess meat production while relying heavily on imported fruit and vegetables – the very foods most critical for human health.^9^ Government subsidies overwhelmingly favour animal farming, leaving arable and horticultural producers with minimal support.^10^ As a result, farming policies fail to align with health, climate or biodiversity goals.

## Why healthcare must lead the transformation

**Fig. 2:**
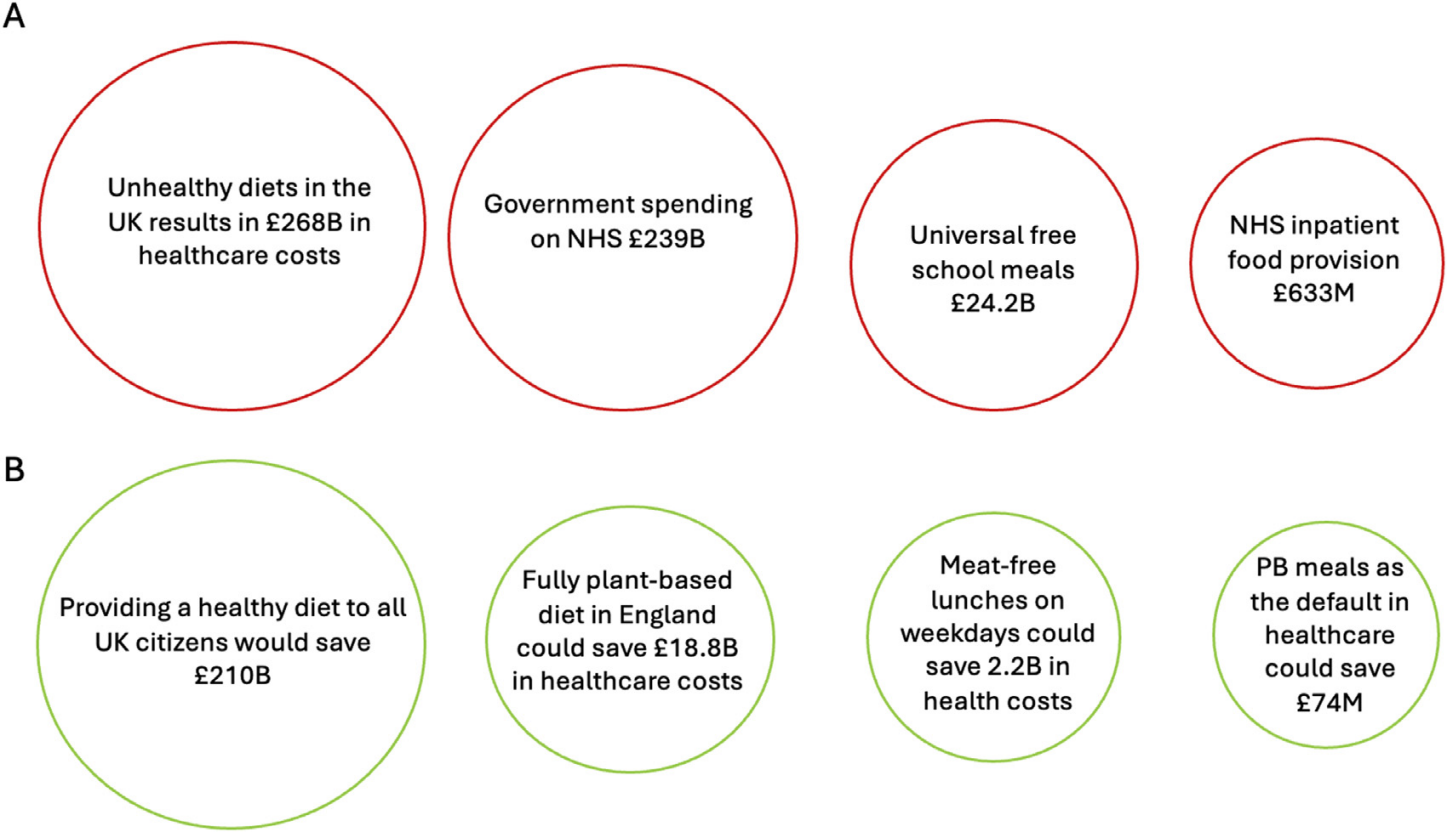
Financial impact of current diets and potential cost savings of a plant-based transition. A: Costs to Government B. Potential savings from a plant-based diet. Figures are annual costs, taken from references 9, 24, Gov.uk, urbanhealthrg.uk, https://www.ohe.org/insight/could-plant-based-diets-transform-health-care-spending/. B, billion; M, million; PB, plant-based.

Prevention should be the cornerstone of any healthcare system, but the NHS is struggling. Chronic disease rates are rising, leaving a widening gap of over 11 years between lifespan and healthspan.^11^ Dietary risk factors are a major driver. The Food, Farming and Countryside Commission reported that unhealthy diets are resulting in a cost of £268 billion annually to healthcare and the economy from health, social and welfare costs and productivity losses, thus surpassing the NHS’s annual budget (Fig. 2).^12^ This staggering cost stems from widespread overconsumption of ultra-processed foods and insufficient intake of fibre-rich fruit, vegetables, whole grains and legumes. This in part is due to the disproportionately low cost of ultra-processed foods compared to healthy whole foods and unregulated advertising and marketing.

Mounting evidence demonstrates that a diet centred around healthy plant-based foods is associated with considerable health benefits, with significant reductions in cardiovascular disease, type 2 diabetes, obesity, certain cancers, kidney failure and fatty liver disease (Table 1). Thus, addressing dietary risk factors and supporting a shift to a plant-based diet has to be at the forefront of the NHS’s prevention strategy.

**Table 1:** Benefits of a healthy plant-based diet.

Disease	Risk reduction
Coronary heart disease^a^	25%↓
Type 2 diabetes^b^	34%↓
Cancer risk^c^	15%↓
Stroke^d^	10%↓
Renal failure^e^	14%↓
Fatty liver^f^	24%↓
Parkinson’s disease^g^	22%↓
Sleep apnoea^h^	17%↓
COVID-19 incidence/severity^i^	10%↓/40↓%
All-cause mortality^j^^,^^k^	10–16%↓

a J Am Coll Cardiol, doi:10.1016/j.jacc.2017.05.047.

b PLOS Med, doi:10.1371/journal.pmed.1002039.

c Int J Cancer, doi:10.1002/ijc.31593.

d Neurology, doi:10.1212/WNL.0000000000011713.

e Clin J Am Soc Nephrol, doi:10.2215/CJN.12391018.

f Clin Nutrit, doi:10.1016/j.clnu.2018.08.010.

g Mov Disord, doi:10.1002/mds.29580.

h ERJ Open Res, doi:10.1183/23120541.00739–2023.

i Gut, doi:10.1136/gutjnl-2021–325353.

j Circulation, doi:10.1161/CIRCULATIONAHA.119.041014.

k JAMA Netw Open, doi:10.1001/jamanetworkopen.2023.4714.

## Diet and planetary health: an inseparable connection

The global food system contributes approximately one-third of greenhouse gas emissions, with more than half generated from animal agriculture.^13^ The UK’s agricultural sector accounts for 30% of the nation’s emissions (including emissions produced by the food that is imported), with 74% of these emissions stemming from red meat and dairy production.^9^^,^^14^ The extensive use of land and other natural resources for raising animals for food makes the UK one of the most nature-depleted places in the world.^15^ Farming animals is hugely inefficient as they require, depending on the species, 2–12 kg feed to produce 1 kg food for humans, with animal-sourced foods only providing 18% of calories globally and one-third of calories in the UK.^16^ Often forgotten is the energy required for farming and thus the reliance on fossil fuels, with a disproportionate amount used for producing food from animals.^17^

**Fig. 3:**
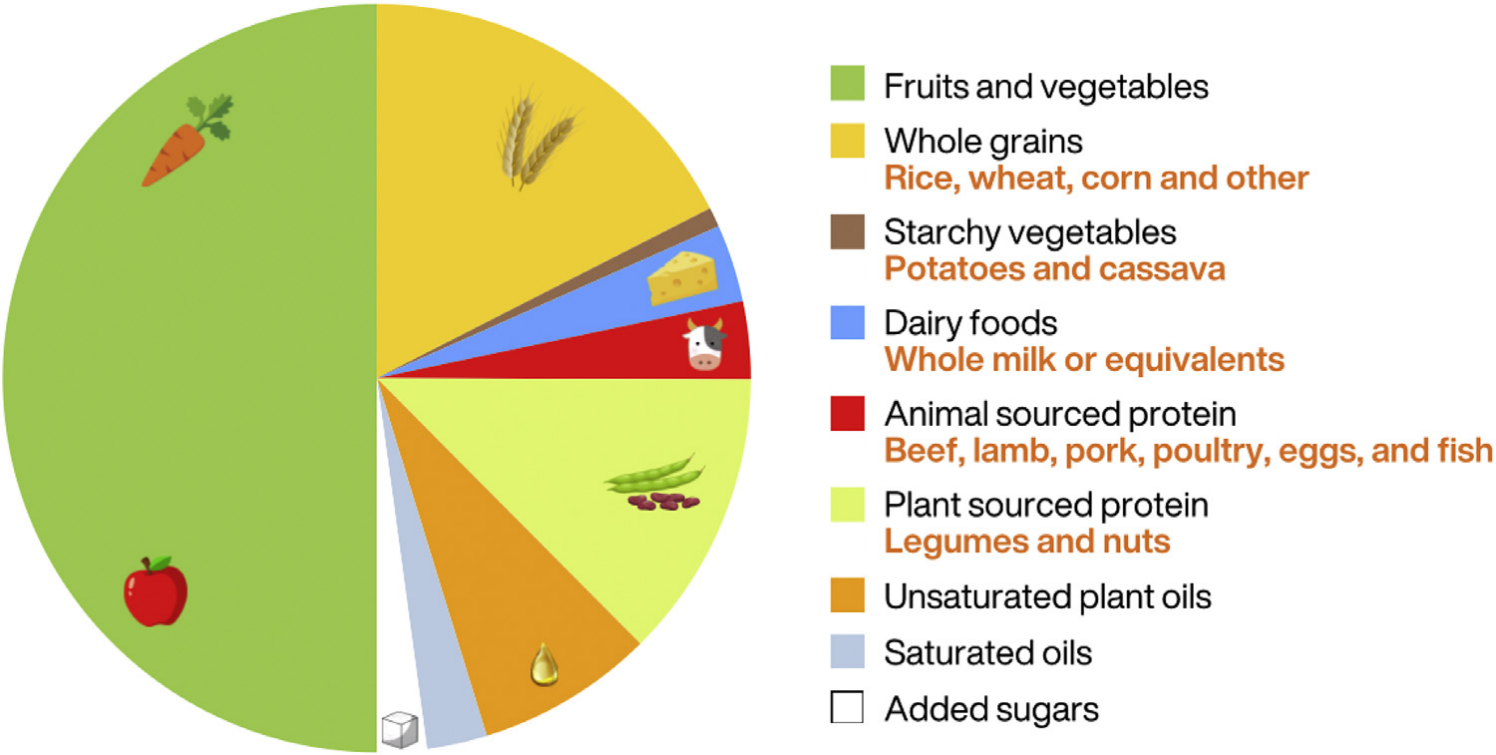
Eat Lancet Planetary Health Diet: An overview of the planetary health plate. By volume half of the plate is filled with fruits and vegetables. The other half is represented as contribution to caloric intake, and consists mostly of whole grains, plant protein and unsaturated plant oils. Reproduced from the Eat-Lancet report.^3^

There is good news. A diet that promotes human health can also support planetary health. As described in the Eat Lancet commission report, shifting to a plant-based diet with at least 85% of energy derived from healthy plant foods is expected to keep the food system within planetary boundaries while at the same time providing healthy food for the entire global population.^3^ The authors devised a reference diet known as the Planetary Health Diet (Fig. 3). This minimises meat and dairy consumption and ultra-processed foods and acknowledges that animal-source foods are not essential. To achieve a sustainable diet, the UK public will have to reduce meat consumption by up to 80% while increasing the consumption of healthy plant foods by up to fourfold.

Eliminating red meat and dairy consumption would have a substantial impact on both environmental and health outcomes.^1^ A fully plant-based transition could free up 75% of farmland, enabling ecosystem restoration and carbon drawdown equivalent to 16 years of fossil fuel emissions by 2050.^16^^,^^18^ Additionally, reducing methane – a potent greenhouse gas produced by ruminant animals – offers immediate climate benefits, as its shorter atmospheric lifespan means that results could be seen within our lifetime.^19^

There are further benefits to health. Globally, 70% of antibiotics are used in animal farming, fuelling antibiotic resistance. Phasing out industrial animal farming could mitigate this risk.^20^ Many new and emerging infectious diseases originate from animal farming. Reducing reliance on animals for food decreases this threat.^21^ Farming also contributes to air pollution, through the generation of ammonia from animal waste and fertiliser use, with around 30% of particulate matter pollution in our UK cities coming from farming, the majority from animal farming.^22^ Faeces from farm animals pollute rivers, killing plant and animal species and making the water unsafe to humans.

A fully plant-based diet has the lowest impact on all metrics of planetary health^23^ and it is reassuring to know that a plant-based diet can also support healthy living at all ages and stages of life, with numerous benefits to cardiometabolic health, longevity and athletic performance.^24^

## Leading by example: healthcare’s role in the transition

Healthcare organisations have a unique opportunity to lead the transition towards plant-based diets. They can normalise plant-based meals within hospitals, using patient interactions as teachable moments. A successful example comes from New York City Health and Hospitals, where plant-based meals have become the default option in 11 hospitals.^25^ This initiative has led to over 50% of eligible patients selecting the plant-based meal, a 36% reduction in food-related carbon emissions, significant cost savings and high levels of patient satisfaction – all without restricting choice.

The success of such programmes hinges on presenting plant-based meals as attractive, culturally inclusive and delicious. Modelling studies suggest that the NHS could save up to £74 million annually by adopting similar practices.^26^ This ground-breaking approach has now been brought to the UK by the grassroots, clinician-led Plants First Healthcare initiative (plantsfirsthealthcare.com) through a collaboration with the non-profit organisation Greener by Default, who spearheaded the New York City initiative.

It is disappointing to witness the current lack of ambition from NHS England’s net zero food team. They have committed to replace beef on menus with meat from deer rather than supporting people to eat more plant sources of protein. Their newly created recipe bank has numerous dishes that include beef (foodplatform.england.nhs.uk). This is despite the fact that all meat from mammals, ie red meat, has been classified as a class 2 carcinogen, probably causing cancer, and is associated with increased risks of cardiovascular disease, type 2 diabetes, dementia and inflammatory conditions.^27^^,^^28^ Our own analysis of hospital menus has shown limited progress in offering environmentally friendly meal options,^29^ despite the NHS’s commitment to net zero emissions.

## Overcoming resistance and misinformation

Healthcare organisations are overly worried about restricting choice or making bold changes to inpatient catering, when our own research shows that if patients are made aware of the detrimental effects of foods such as red and processed meat to their health and the planet, most are supportive of removing these from menus.^30^ An independent survey of 2,000 nationally representative people in the UK commissioned by Plant-Based Health Professionals UK showed that most people did not consider hospital food to be flavourful or healthy and thought that it needed to change.^31^ In addition, almost one-third of respondents said that they would be supportive of a 100% plant-based menu, while a majority were supportive of normalising plant-based dairy alternatives. So in fact, the public are open to what some consider to be radical change.

## Policy and practice: the way forward

Numerous guidelines and policy documents already highlight the benefits of a plant-based diet for health and the environment.^32^^,^^33^ It’s time to act on this knowledge. Simple measures, such as removing red and processed meat from hospital menus, implementing plant-based defaults, and amplifying advocacy efforts can drive significant change. By embracing these actions, the healthcare sector can accelerate a transition towards a sustainable, plant-based food system. This shift is not merely an ethical imperative but a practical solution to the pressing challenges of our time.^34^

Recommendations for healthcare leaders and organisations (adapted from the UK Health Alliance on Climate Change food policy report).^32^

• Support hospitals to normalise plant-based meals as the preferred option for patients and staff.
• Support the replacement of red and processed meat on menus with plant sources of protein and implement plant-based dairy alternatives as the default option.
• Commit to fully plant-based catering for meetings, events and conferences.
• Promote education and awareness of the benefits of a plant-based diet.
• Endorse the Plant-Based Treaty.
• Use your trusted status to influence policy and lobby decision makers to support the transition to a plant-based food system.
• Divest from banks and companies that support and finance animal agriculture.

## Declaration of generative AI and AI-assisted technologies in the writing process

During the preparation of this work the author(s) used ChatGPT 4o in order to make the manuscript more readable. After using this tool/service, the author(s) reviewed and edited the content as needed and take(s) full responsibility for the content of the publication.

## Funding

This article did not receive any specific grant from funding agencies in the public, commercial, or not-for-profit sectors.

## CRediT authorship contribution statement

**Shireen Kassam:** Writing – original draft, Conceptualization. **Laura-Jane Smith:** Writing – review & editing, Conceptualization.

## Declaration of competing interest

The authors declare the following financial interests/personal relationships which may be considered as potential competing interests: Shireen Kassam reports a relationship with Plant-Based Health Professionals UK that includes: board membership. If there are other authors, they declare that they have no known competing financial interests or personal relationships that could have appeared to influence the work reported in this paper.
